# Improvement of Kafka Streaming Using Partition and Multi-Threading in Big Data Environment

**DOI:** 10.3390/s19010134

**Published:** 2019-01-02

**Authors:** Bunrong Leang, Sokchomrern Ean, Ga-Ae Ryu, Kwan-Hee Yoo

**Affiliations:** Department of Computer Science, Chungbuk National University, Chungdae-ro 1, Seowon-Gu, Cheongju, Chungbuk 28644, Korea; bunrongleang@chungbuk.ac.kr (B.L.); chomrern@chungbuk.ac.kr (S.E.); garyu@chungbuk.ac.kr (G.-A.R.)

**Keywords:** Hadoop ecosystem, public-key cryptography, data processing, data streaming, real-time analysis, secured PLC sensing data

## Abstract

The large amount of programmable logic controller (PLC) sensing data has rapidly increased in the manufacturing environment. Therefore, a large data store is necessary for Big Data platforms. In this paper, we propose a Hadoop ecosystem for the support of many features in the manufacturing industry. In this ecosystem, Apache Hadoop and HBase are used as Big Data storage and handle large scale data. In addition, Apache Kafka is used as a data streaming pipeline which contains many configurations and properties that are used to make a better-designed environment and a reliable system, such as Kafka offset and partition, which is used for program scaling purposes. Moreover, Apache Spark closely works with Kafka consumers to create a real-time processing and analysis of the data. Meanwhile, data security is applied in the data transmission phase between the Kafka producers and consumers. Public-key cryptography is performed as a security method which contains public and private keys. Additionally, the public-key is located in the Kafka producer, and the private-key is stored in the Kafka consumer. The integration of these above technologies will enhance the performance and accuracy of data storing, processing, and securing in the manufacturing environment.

## 1. Introduction

Recently, some manufacturing industries throughout the world have started to use data to record their work activities and factory behavior. Before technology growth, hand-writing was used to record data in companies or factories, but this method is inefficient when there is a large amount of data. Moreover, it cannot assure the security of the data and costs a lot of money to maintain. There have been many innovative ideas to overcome these limitations, and there are plenty of researchers that deal with Big Data [1,2,3,4,5]. In particular, Big Data has been used in a state-of-art framework for tremendous amounts of storage for data and intelligence in many research, including manufacturing, smart city, smart farm, and medical systems [6,7,8,9,10]. This innovative idea can practically solve a lot of modern problems.

In recent years, technology has grown quickly, and the Big Data industry continues to upgrade to the latest technology and new innovative ideas. There are a lot of industries that apply this concept to upgrade their systems such as healthcare, manufacturing, and social network in a Big Data system. The manufacturing industries are upgrading to smart factories [2,3,4]; smart factories are a potential topic being researched in the manufacturing industry research area. There are many researchers aiming to overcome certain issues in factories such as processing log data, analyzing sensing data, and storing tremendous amounts of machine log data. Sensed data or machine log data are generated every second, so this is a critical issue related to the storage of data in the Big Data platform. There are plenty of methods that can be used to store vast amounts of data, such as Hadoop Ecosystem [5] and other commercial databases. However, some giant companies use their own data store and technologies, which are unavailable to open source users.

Nowadays, manufacturing Big Data systems is popularly done using the Hadoop Ecosystem for storing and processing data, since Hadoop is open source and allows for many software libraries or server libraries on it. The designation of the Hadoop ecosystem is very essential for Big Data engineers to set up the Big Data environment. The Hadoop file system comprises some software to work with the data. Hadoop distributed file system (HDFS) is mostly used to store data as a file, but Apache HBase [11] is a distributed or NoSQL database that is famous and can be used with the Hadoop system. Interestingly, the ability to handle trillions of data points is a feature of the Hadoop platform.

Moreover, joint ventures are very critical in allowing all parties to work together in the same environment, and is the key point of data transmission. There are some big companies and industries investing large amounts of money into university research projects in order to enhance their production process. Similarly, manufacturing spends a lot of money in building smart factories and analyze the machine log data with the university. For instance, some factories cooperate with research institutes and universities to build Big Data platforms including data analysis. Therefore, data streaming and transmission are necessarily required in such projects. According to the results of recent papers [12,13,14,15], it is believed that the Apache Spark and Apache Kafka could be one of the best choices in terms of the real-time data streaming and transmitting. We can use these technologies to overcome the problems between the factory and other parties, so that the data that are transmitted from the factory will be consistent with every party. All of this installation environment is integrated to work together, as a Hadoop ecosystem.

Significantly, the data for this study are generated from the programmable logic controller (PLC), which is defined as a computer machine without display and keyboard. It is used as an industrial control in smart manufacturing. For example, the PLC is able to provide the status of the machine such as temperature, alarm signal, and operation state. From this information, it is essential to achieve the efficiency of business operation [1,2,3,4,5]. The advanced technology of hardware development enables us to afford, and make use of, the functionality to achieve the goal of adopting industry revolution 4.0.

In fact, programming alone cannot deal with all the issues in the complex environment of production lines in shop flow. So far, questions have been raised about the safety of data gathering program and led to an examination of the effects of data encryption and decryption.

With this mind, the technique of cryptography has been chosen to secure data transmission. In order to do that, the public/private key encryption and description have been put into practice. First, the data collection layer works directly with the PLCs devices to get the sensing data and publish the data to the Kafka producer. To secure the data transmission over the network, we are aware that data encryption and decryption could be served as the tool to protect from cyber threatening. To do so, a public/private key is introduced to apply on Kafka messages. Basically, there are two major steps, which are encryption and decryption. Firstly, the messages are encrypted with the assigned public key by the Kafka producer. Next, the encrypted messages will be transmitted through the network by internet protocol. Eventually, the decryption is applied by the Kafka consumer to decrypt the message. Notably, the process of encryption and decryption will provide secured data transmission over network. 

The paper consists of nine sections. Section 1 introduces the general concept of real-time data transmitting and processing in the firms. Section 2 presents the related study. Section 3 gives an overview of the system architecture. Section 4 highlights the designation of data store regarding the Hadoop and HBase. Section 5 describes the data streaming and processing using Apache Spark and Kafka. Section 6 examines the recommended partition and thread to achieve higher performance. Section 7 addresses the secured data transmission using public/private key cryptography. Section 8 suggests the experimental result. Section 9 summarizes the paper and indicates future work.

## 2. Related Studies

In this section, there are several ideas that describe the Big Data environment, including real time data streaming, and the Hadoop ecosystem. All these concepts are essential to pave the way for understanding and contributing to the proposed method in this paper. Therefore, the related studies of this research are given below.

Firstly, a single processing is not a promising technology for working with large amounts of data such as machine log data, sensing data, and so on. However, the distributed system (Cluster) is a promising method in Big Data research for handling vast amounts of data (integrating the number of server machines for parallel processing). For instance, the Hadoop Distributed File System provides a cluster mode environment for storing large amounts of data. In order to do this, HDFS splits file sizes that exceed the amount of block size configuration, which by default is 64 Meg-Bytes (MBs) [16]. This number can be changed in the future, and there are a lot of configurations available in the configuration file. In addition, working with cluster mode contains many software plugins such as Apache Spark for data processing, Apache HBase for NoSQL database, Apache Kafka for data transmission, and Apache Zookeeper for state coordination.

Secondly, the design and implementation of storage and data processing use the Hadoop as the file system for partitioning big file sizes into smaller pieces, as proposed by Kim [17]. This method has been integrated with the cluster of Kafka for real-time transmission and by using the Hive as the data warehouse. Apache Hive [18] is a data warehouse and also the appropriate integrated engine with Hadoop for storing the log data and historical data. Son et al. [19] proposed a method for anomaly detection for big log data, his proposed method used the Hadoop ecosystem integrated with Hive as a data store to interact with the machine learning algorithms. With Hive, it is very easy to load and query the data, since it provides the SQL syntax for communicating with the Hive data warehouse.

Thirdly, in a Big Data platform, data transmission is a necessary technology for sending data from the factory to other locations (cross-platform) [20]. Many researchers have implemented these technologies, some of them including ZeroMQ [21], ActiveMQ [22], RabbitMQ [23], and Apache Kafka [24]. Ayae et al. [25] proposed a method for transmitting data via Apache Kafka, which provided a high throughput data ingestion system. Apache Kafka can adapt to many kinds of data and to transmit video data after analysis. In order to build upon the performance of his proposed method, Apache Spark was employed for video data processing. Similarly, machine log data was generated rapidly, so transmitting the sensing data requires reliable technology, D’silva et al. [20] proposed a method for transmitting and processing the IoT historic sensing data, that integrated with Dashing Framework, to visualize the historical data on a graph. In the data transmission phase, security is of concern during sending and receiving the data from the Kafka server. Therefore, security levels must be ensured. Griotti et al. [26] proposed a method to mix public and private key for data encryption and decryption for a wireless sensor network, in which the data is decrypted before being sent through the wireless network.

Fourthly, there are a few Hadoop ecosystems which have been applied to some factories, but it still raises some limitations. The above studies did not encrypt and decrypt the data or messages sent through the Kafka server. Therefore, some data security problems might occur that can be exploited by anonymous hackers. Moreover, the Kafka stream size might be limited due to the pipe size of the Kafka server, so public-key cryptography is a good solution for reducing the Kafka message size. In addition, we combined a lot of technologies to work with Big Data manufacturing systems such as public-key cryptography, Apache Kafka, and Apache Spark to make a real-time transmission secured PLCs sensing data.

Finally, our system was developed to transmit data from PLCs to a database server securely. We used Apache Kafka for real-time data streaming. In Kafka, there are some additional features, such as partitioning and threading. Since there are a lot of messages from the PLC devices, Kafka partition was applied to separate the task of storing messages. Multi-threading was used to handle parallel processing of sending and receiving messages, multiple partitions are fited to multi-threading. In addition, to accelerate data processing, we used Apache Spark for processing, analyzing, and cleaning the data. Moreover, we also considered data security when the data was sent from PLCs to the database server through the internet. We used public/private key cryptography to encrypt and decrypt the messages. In our system design, we located the public key at Kafka producer for data encryption and the private key was located at the Kafka consumer for data decryption.

## 3. System Architecture

**Figure 1:**
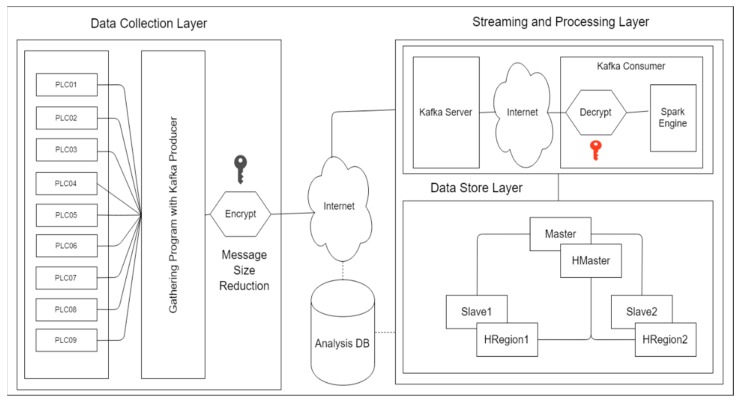
System Architecture and Flow.

In this study, we proposed a Big Data environment in manufacturing for supporting Big Data platforms. In this research, we design a Hadoop ecosystem integrated with Apache Kafka. Moreover, in this section, we divided the whole system into three main parts as shown in Figure 1: A data collection layer, a streaming and processing layer, and a data storage layer.

First, the data collection layer is a layer that works directly with the PLCs devices to get the sensing data and publish the data to the Kafka producer. In this proposed method, we used a public/private key for data encryption, the Kafka producer includes a public key for encrypting the message and also to reduce the message’s size and private key located in the Kafka consumer. The message was sent through the network to other locations and temporarily stored in the Kafka broker (Kafka server). The Kafka consumer was notified that they have received a new message. Secondly, the Kafka consumer included a private key for decrypting and extracting the message and carrying out the real-time data processing via Apache Spark cluster, so all the messages had to be cleaned. Lastly, the data store layer contains the HDFS and HBase, which will store the data. This architecture was installed as a cluster-based one that contains one master and two data nodes, as does HBase. Moreover, we use the MariaDB [27] as an additional database schema to store the basic configuration of the system and the basic statistical analysis. All data are collected directly from many PLCs. The data contains machines status information, such as alarm, run, wait, stop, and manual, and product information, such as values of process variables. Basic statistical analyses on these data are performed such as mean, min, max, frequency. Overall, these three layers have to work together in order to empower the Big Data platform in the manufacturing environment.

## 4. Designation of Data Store

In this proposed method, Apache Hadoop and HBase have been selected as data stores. Both are distributed applications that enable users to work with large datasets. Moreover, Hadoop and HBase are the best combination for large-scale data [12,28,29,30], such as sensing data and social network data. In this configuration, we set it up to work with PLCs sensing data, which is generated every second in the factory. Hadoop version 2.7.3 had to be used in this research project, and in this version, there was a resource manager for managing the resources in the system, called Yet Another Resource Negotiator (YARN). In addition, the Hadoop MapReduce framework had to be used as a default configuration of the Hadoop system to enable performance of multiple tasks. Likewise, HBase was also a distributed application (database) that worked well in combination with Hadoop for scaling datasets, and there were a few slave nodes which could be controlled by a master node.

**Figure 2:**
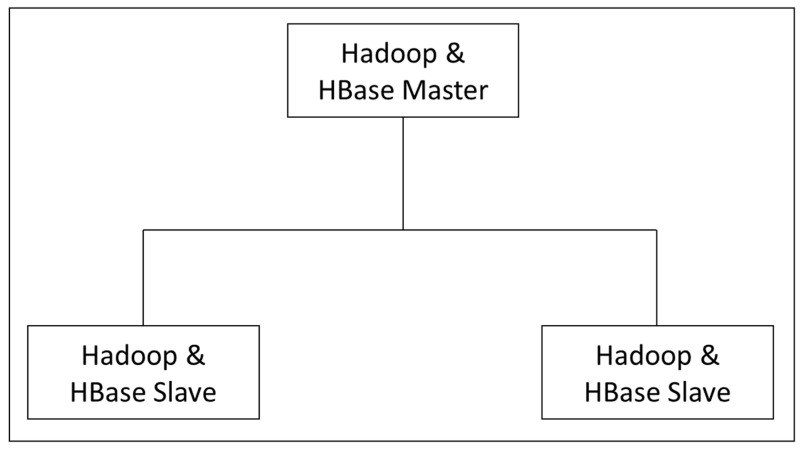
Hadoop and HBase Cluster.

Figure 2 shows the distributed mode of Hadoop and HBase for storing sensing data, we used the master server personal computer (PC) for Hadoop and HBase master since this server PC consists of the high specification, which is good for performing many tasks simultaneously. These two data stores have been integrated so as to scale the data using a column-based oriented, and efficient, retrieving method.

**Figure 3:**
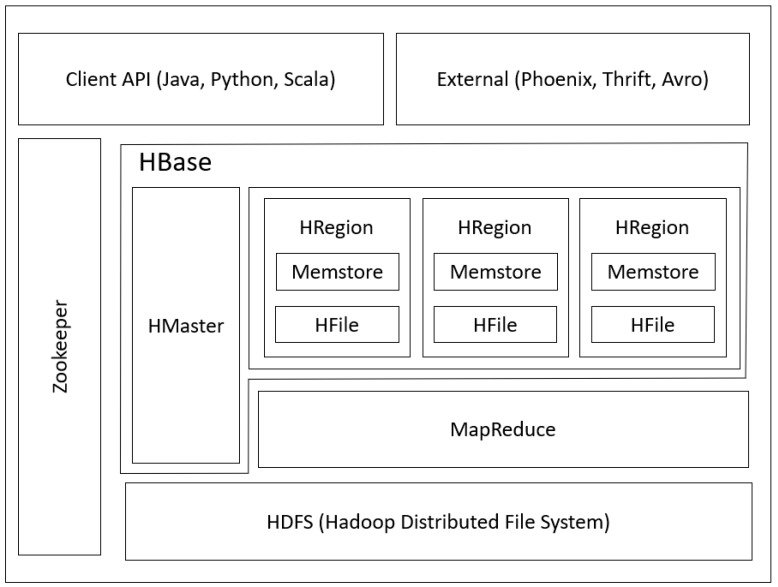
Design Hadoop Ecosystem.

As shown in Figure 3, HBase runs on top of Hadoop and is coordinated by Zookeeper (Zookeeper is a state coordinator of HBase, Hadoop, and other applications). In the Hadoop ecosystem architecture, Hadoop distributed file system (HDFS) is located at the bottom, and is similar to the file system for managing and storing the file. Above the HDFS is the Hadoop File System API for interacting with the file in HDFS. To facilitate the data querying in HDFS, HBase has emerged. Normally, HBase is located on top of the Hadoop and HBase can run as a cluster also. HBase is used as an interface for interacting with the HDFS to retrieve the data, so that the user does not need to work directly with HDFS’s command, otherwise we can use HBase query to retrieve the data. The client side can use a variety of external applications and programming languages to query the data from HBase, such as Python, Java, and Scala.

## 5. Data Streaming and Processing

There are many kinds of data streaming applications, but Apache Kafka was selected as a streaming pipeline in this research. As Kafka is a message queue application, it can perform real-time data transmission, including a new data processing library in the latest version. That is to say, in order to achieve a more efficient application than a Kafka data processing library, Apache Spark cluster mode was installed as the data processing engine and works closely with the Kafka consumer. We used the stand-alone mode of the Kafka application. In the factory, there is a large amount of sensing data generated every day, so multi-threading and some Kafka properties have to be applied. First, Kafka offset is a significant property in the Kafka consumer, and there are three value types of offset property: The earliest, latest, and specific index [31]. In this case, the earliest was selected as a property since we considered we would lose data, so even if there is an interruption during the streaming period, we could still start-up the program and obtain the oldest messages from the Kafka broker. Generally, the messages were not immediately removed from the server. The main reason of doing that was to make sure even if the Kafka consumers failed, Kafka Broker would keep the messages that were sent from Kafka producers. In this case, when the Kafka consumers restarted, they could receive the data without losing the data. When our experimental research was performed, normally, the system could not fail for more than three days in the real world. So, we suggest temporarily store the data for three days.

**Figure 4:**
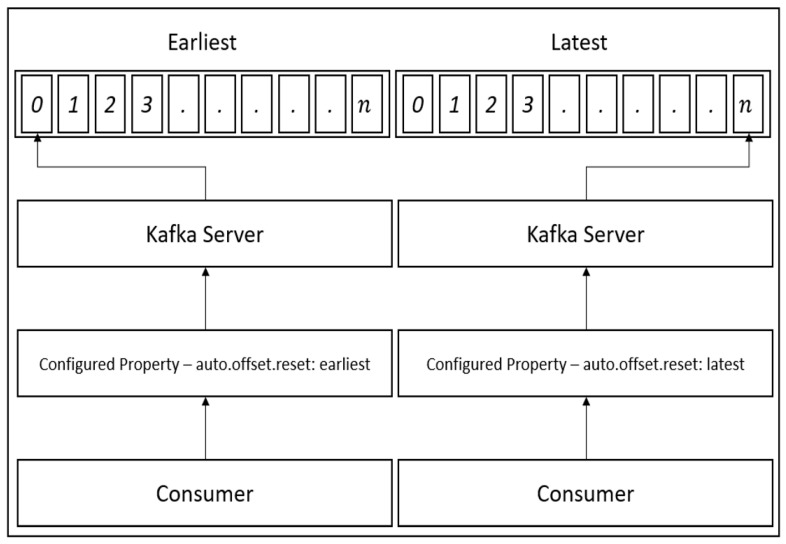
Kafka Offset Configurations and Properties.

Figure 4 depicts the offset selection in the consumer, where the earliest property is the best choice to prevent the loss of data where there is an interfering event from external sources. However, there is also a disadvantage in performing this offset, as the consumer will read duplicate messages that have already been read by the prior consumer group. Therefore, to overcome this drawback, the validation of the duplicate messages is conducted by the consumer. As a result, there is no longer a major issue reading the messages and the performance of validation is also fast enough.

Kafka contains a useful feature for scaling Kafka consumers to ensure that our program can work on a tremendous amount of data, and we have to ensure that the message can be real-time. This is called partition [32]. The partition was created when initializing the topic, and we could define the number of partitions, where a large number of partitions were illustrated in the form of the complex program, as is the only way to scale the Kafka consumer.

**Figure 5:**
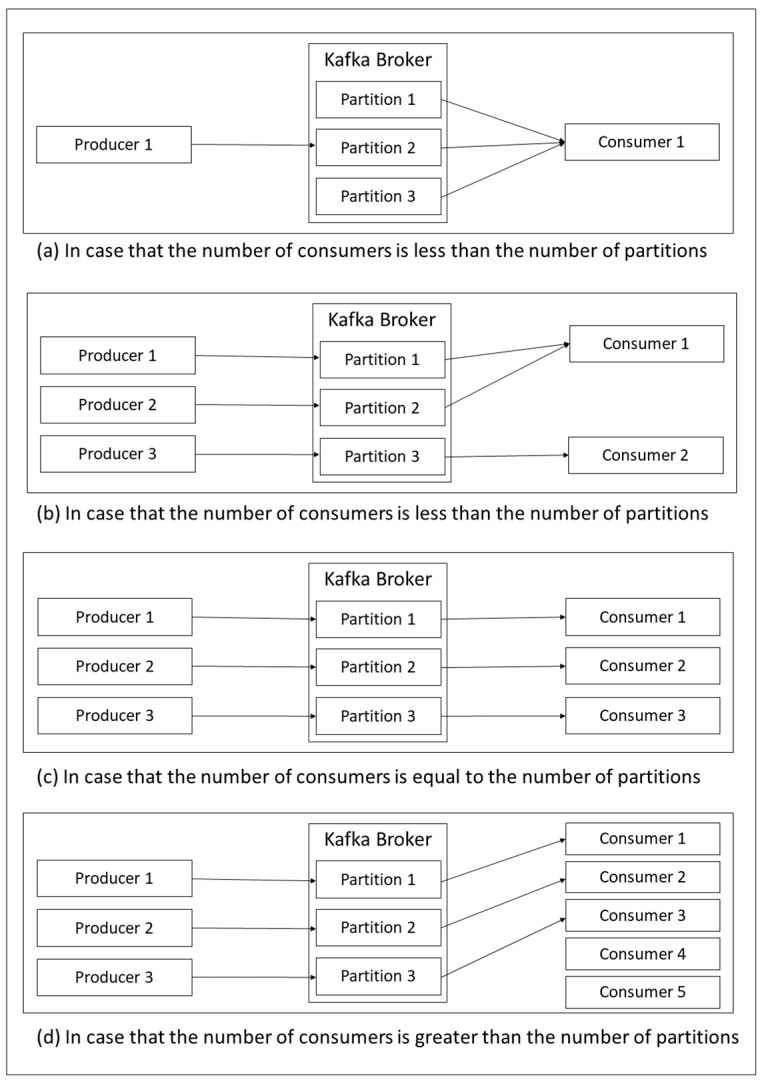
Partitioning in Kafka Consumer.

There are several styles of consumers and partitions that we are faced with in the real world, so the partitions have been assigned to different consumers depending on the number of existing consumers. Figure 5 illustrates the inconsistencies and consistencies between consumers and partitions. As shown in Figure 5a,b, if the number of consumers is less than the number of partitions, ones of all consumers will be assigned more tasks than others. On one hand, if the number of consumers is equal to the number of partitions, the assignment will work perfectly as presented in Figure 5c. On the other hand, if the number of consumers is greater than the number of partitions, there will be unused consumers because there is no partition assignment from the Kafka broker as illustrated in Figure 5d.

Lastly, in order to improve the Kafka performance and reduce the server’s random access memory (RAM) and tasks, multi-threading is performed. Using multithreading provides time-sharing and memory-sharing [33], since one process contains a lot of threads, so RAM has to be reduced. In our system design, we ran one process, which contained six threads because the factory is made up of many lines, and one line supports six to seven machines. As a result, we could define the number of threads based on this number. These threads handle a vast amount of sensing data that are transmitted via the Kafka producer.

**Figure 6:**
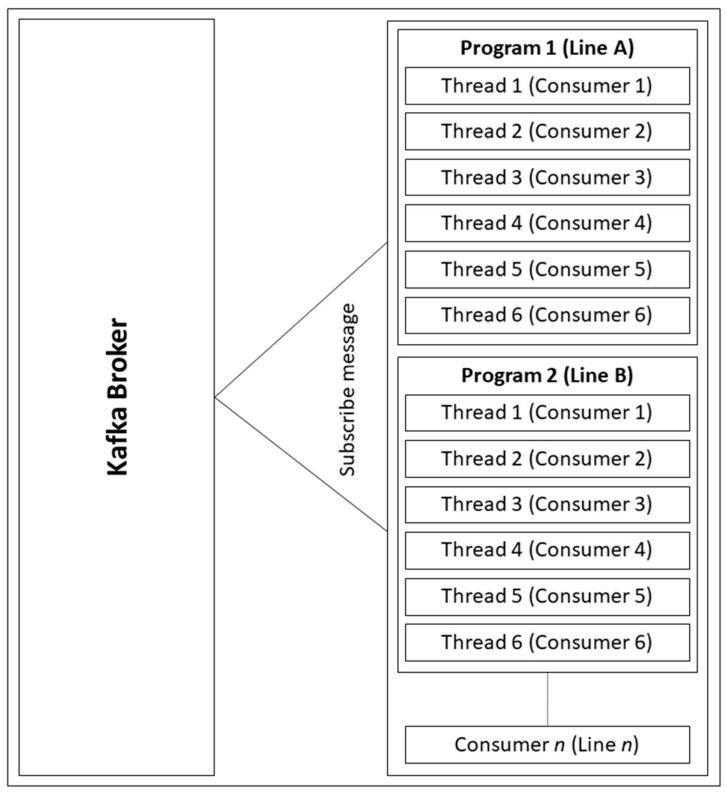
Multi-threading in Kafka Consumer Program.

Figure 6 shows the time and memory sharing using multi-threading (lightweight), a Kafka program represents one line in a factory. As one line normally consists of six machines, six threads are initialized to subscribe the message from the Kafka broker. In this case, one program is equal to one line (factory) or one group (Kafka), so if there are n lines, there will be n programs waiting to receive the messages.

The gathered data from PLCs, which were stored in the Database, such as MariaDB or Hadoop in a server side, need to be processed and analyzed to detect the outlier of the product variables, and to give predictions of machine faults and product faults. Apache Spark is a distributed processing engine application that can be embedded with Hadoop and Kafka to make a real-time processing and analyzing application. Here, we used Spark as a processing engine and installed as a cluster mode (one master and two data nodes). This processing engine is closely related to the Kafka consumer, and the role of Spark is used for cleaning the data when the consumer receives messages from the producer. Since the received messages were in JavaScript object notation (JSON) form, we had to extract the JSON object and to prepare the data for the database. Moreover, Spark performed a basic statistical analysis of the raw data and transferred them to the database. In addition, Spark was also a good application to integrate with HBase and make an efficient query. We tested the Spark query from the HBase database, and the resulting performance was good, since Spark is memory processing-based, its performance is better than MapReduce [34]. The speed increased when the Spark cluster was larger and the server PC’s specification was very high. Spark query plays an essential role in data retrieval and visualization in the graphic user interface (GUI), where data driven document (D3) is used as a visualization library. Lastly, Spark provided a very useful library in Java, Python, and Scala to work with a Spark cluster server on the client side and also provided a useful collection framework—resilient distributed dataset (RDD) [35].

## 6. Recommended Partition and Thread

As mentioned in the previous section, it is clear that the two effective methods of the partition and the thread can work efficiently in terms of improvement of the Kafka streaming data in the manufacturing environment. Apart from that, using thread without calculation can cause a lot of problems, such as memory leak, CPU error, and so on. Calculating or limiting the number of threads in the consumer program should be considered instead. We proposed a method to calculate the number of partitions and thread that works well with both PLCs and server PC. This solution can prevent CPU error and memory leak, including the non-real-time data gathering. The number of partitions and consumers should be equal, but in the consumer, multithreading is performed, so one thread is equal to one consumer. Finding the number of the partition can lead to finding the number of threads.

We can assign one thread to work with two partitions (two machines), so we can define the number of threads with the below formula by using a ceiling operator.

(1) $$number\;of\;thread=⎡\frac{number\;of\;PLCs}{2}⎤$$

This formula can express the number of threads or partitions divided by two or more than two, depending on user preference. However, we recommend two as a denominator in the above formula, since a large number can cause problems in the server and data transmission. If the number of PLCs is an odd number, the result will be a decimal, but the result cannot be a decimal, so we use would a ceil function to round up the result.

**Figure 7:**
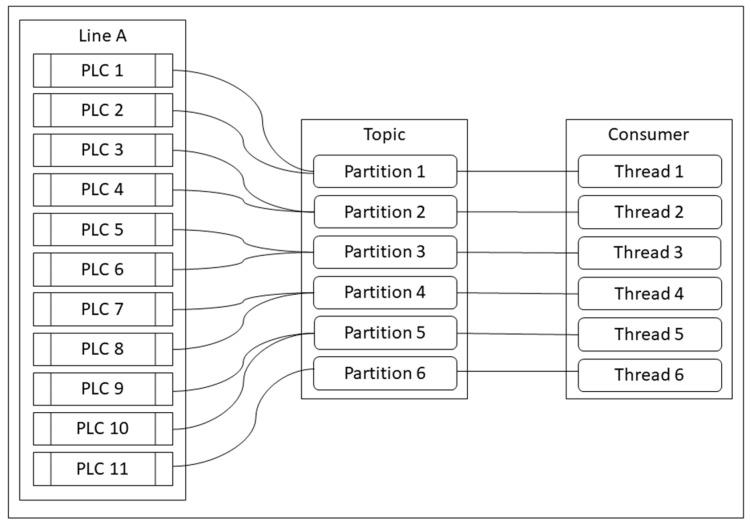
Recommend the number of partitions and threads.

Figure 7 shows the eleven PLCs (odd number), and we recommend the appropriate number of partitions and threads with these PLC numbers using the above formula and ceil function. Thus, eleven PLCs will use six partitions and six threads to consume the data, so the original result will be 5.5, but the ceiling function has to be performed to round up the result to six, so one consumer program will contain six threads to gather and transmit the data from PLCs. In short, eleven PLCs will use six threads in a consumer program and a topic consists of six partitions, so one thread can handle at least two PLCs. In cases in which there are many lines, there will be many consumer programs and the number of the thread will be calculated based on the number of PLCs in one line.

## 7. Secured Data Transmission

In this proposed method, public-key cryptography has been selected as an encryption and description method. There are many appropriate security methods [36,37,38,39], but public/private key cryptography is one of the good options for us to encrypt/decrypt the data [39]. The reason is as follows: To provide more secure data transmission over network and data management in several servers, we assigned a public key to Kafka producers in a manufacturing factory and a private key to Kafka consumer in each server. The Kafka producers try to encrypt the gathered data with a public key, which is gathered from PLCs of machines in the factory and send the encrypted data to more than one server over network. And then the Kafka consumer in each other server can try to decrypt the data with each private key. This method provides two keys, public and private, for encrypting and decrypting, respectively. This method uses the public key to encrypt the message and the private key to decrypt the message. In this real-time streaming, Apache Kafka is used to transmit the data from PLCs to the data store. Therefore, in this case, the public key is in the Kafka producer for encrypting all the messages that will be transmitted to the Hadoop server. With this encryption, we can be sure that all messages are secured, even the messages that could be attacked by hackers. In addition, the size of the message will be reduced after performing this encryption method. Similarly, all of the secured Kafka messages are stored in the temporary data store of the Kafka broker. These messages will be consumed by the Kafka consumer that contains the private key for decryption. Following decryption, all messages will be turned to origin messages, and the Spark engine is called to process this bunch of data.

**Figure 8:**
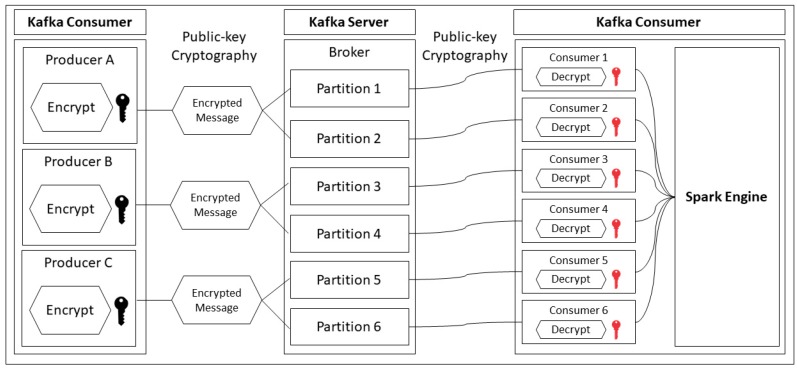
Secured Kafka messages by using public/private key cryptography.

Figure 8 shows the whole system architecture of data security between the producer and consumer. As mentioned in the above section, the key at the producer (left side) is a public key that has been copied from the data store server for encrypting. By contrast, the key at the consumer side (right side) is the private key for decrypting. The private key cannot spread out to other parties or external systems, since this is a secret key, as well as the only key that can decrypt the message.

## 8. Experimental Results

This proposed method was designed and implemented simultaneously. Kafka implementation used six programs to grasp the messages from the PLC.

**Figure 9:**
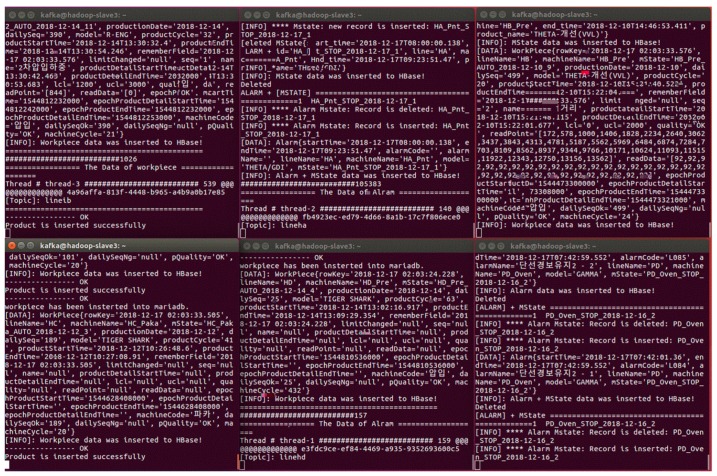
Six programs for grabbing sensing data.

Figure 9 shows the Kafka consumers which consume the sensing data from PLCs. We used six terminal tabs to represent six Kafka programs. Each program consisted of six threads to calculate and process the data more efficiently. All the messages were decrypted, cleaned, and pre-processed before being sent to the Hadoop data storage. That is to say, the Kafka consumers were used with multiple threads and partition. This led to an improvement in the speed of streaming data so it is more effective and efficient.

**Figure 10:**
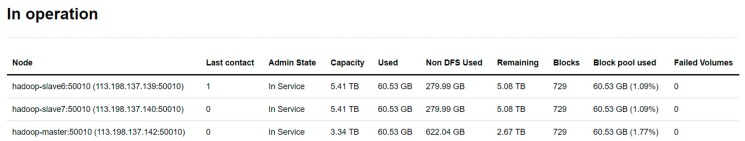
Kafka Producers with partitions and threads (gathering the sensing data from programmable logic controller (PLCs)).

Figure 10 gives an overview of the data node information. Meanwhile, we can use this information to check the details of each data node. Once again, this information provides useful figures, such as the hard disk usage in each data node server as well as the number of blocks being used. Moreover, the interface also worked as the dashboard to give us a chance to check that the remaining disk space of every node was user-friendly.

**Figure 11:**
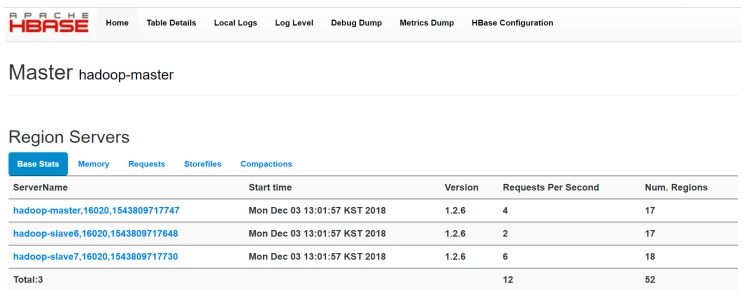
Hadoop Cluster Installation and Information.

Another key thing to remember is that the HBase served as a database for managing the data in the Hadoop data storage since HBase is a distributed database. This means that we were able to design an HBase cluster on top of the Hadoop which contains HMaster and two HRegions as shown in Figure 11.

We also considered the system performance would work with the vast amount of data generated by the machine. The below figures illustrate the result of system evaluation and testing.

As mentioned in the above section, if there are many partitions, the speed of consuming messages is also high. Since the large number of partitions should be appropriate with a large amount of sensing data, the number of partitions and consumers should be equal, because one partition will be considered to only work with one partition. To put it another way, we limited our partition to fit with the size of our data. If our data was too small and we assigned a high number of partitions, it would become a useless partition.

In fact, we can use the Kafka stream API to process and clean our data-set, but this might lead to some problems in the future when there are substantial amounts of sensing data. Therefore, the Spark Engine is considered as a processing engine to clean up the sensing data. One Kafka message consists of a lot of records, and there are many machines that transmit the data simultaneously, so the message will be large. The big cluster size of Spark can reach the low elapsed time due to the fact that the distributed system can handle a vast amount of many tasks simultaneously.

**Figure 12:**
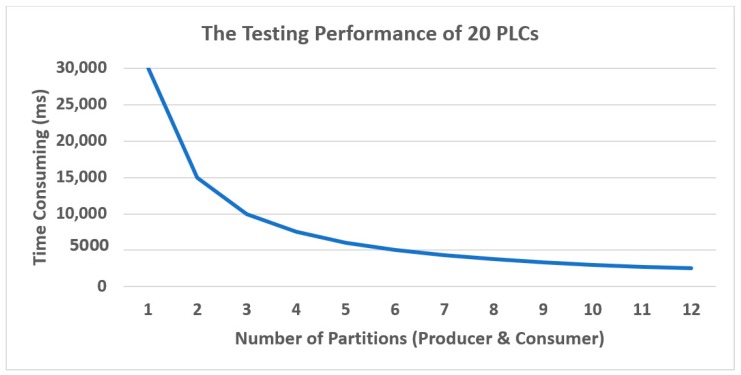
The Testing performance of PLCs and partitions in Kafka.

Figure 12 shows the time consuming of using the number of partitions, if we use the big number of partitions, the time consuming will be reduce. To give the number of partitions, we should consider about the number of messages and PLCs that we have if the number of messages and PLCs are small, the number of partitions should not be big. It will be useless if the message and PLC are not fit to number of partitions and the time-consuming will not reduce too much as in the Figure 12. 

**Figure 13:**
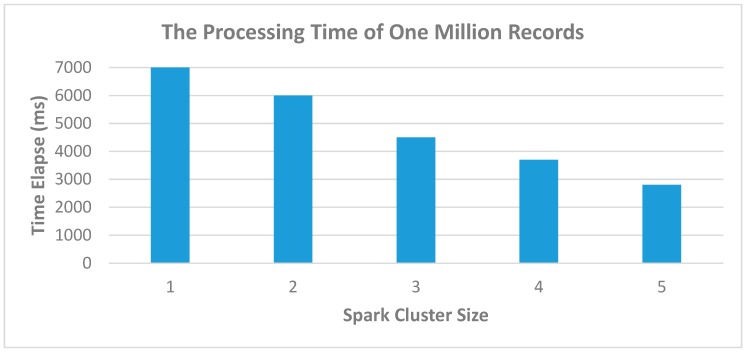
Using Spark Cluster to improve processing time.

Figure 13 depict the time elapse of Spark cluster, the concept is similar to Figure 12, the big cluster will reduce the time-consuming, but the number of clusters should not too big, it is better to appropriate with the number of messages. Even there is a big cluster, but the messages are small, we cannot use all the resource of Spark cluster and the time-consuming is not reduce too much also. 

**Figure 14:**
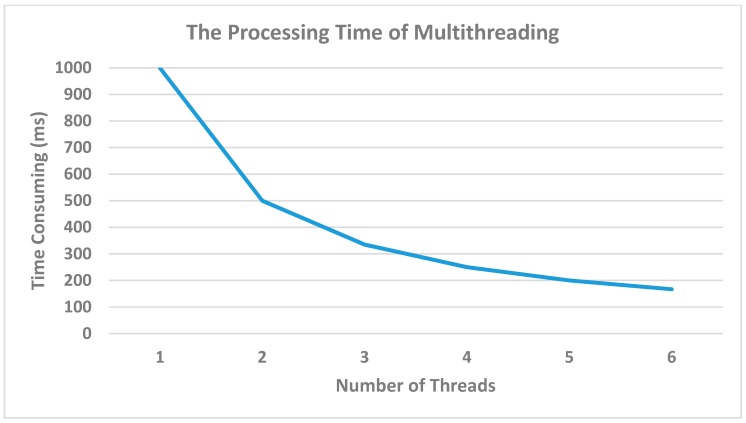
Using Multithreading in Kafka Consumers to reduce the time-consuming.

Figure 14 illustrates the time-consuming nature of using single and multiple threads; as mentioned above, there are six programs used to transmit data. Each program used six threads to accelerate the performance. Using one thread is considered low performance; on the other hand, using six threads is a better solution since one line contains around six to seven machines, so we can convert this number to the number of threads. However, the larger number of threads will be useless if there is a smaller amount of data. We considered the best number that is suitable to our current process.

Figure 14 illustrates the time-consuming nature of using single and multiple threads as mentioned above, there are six programs used to transmit the data. Each program uses six threads to accelerate the performance. Using one thread is considered to be low performance; on the other hand, using six threads is a better solution, since one line contains around six to seven machines, so we could convert this number to the number of threads. However, a larger number of threads would be useless if there is a smaller amount of data. The best number that is suitable to the process should be considered. 

A few studies have indicated how to work with multiple threads and partitions in terms of data streaming and transmission in Apace Kafka and Apache Spark, yet this paper is apart from that by looking at the comparison between the absence of both thread and partition and the participation of threads and partitions. Our experimental results can be seen as in the Table 1. There are some features are needed to take into account such as the duration of time consuming, number of deployed consumers, number of handled PLCs per program, scalable, and parallel processing. AS for dataset, the data is generated by shop flow in a factory which located in Republic of Korea. The size of data is over million records. In addition, this architecture has been deployed on a Linux Server with the specification of CPU, Dual Intel Xeon Processor E7 Family; RAM, 32GB; HD, and 500 GB SSD to perform this method more effectively.

Table 1 provides the information to showcase the efficiency in terms of performance by comparing the application without applying thread and partition and the implementation with thread and partition. To give an insight into what the table means, let’s look at the first column that consists of many measurement features. The first feature is time consuming, it shows how many milliseconds the Apache Spark takes in order to finish streaming the data from the server. The second feature is the number of deployed consumers which is created by client side. The third feature is about the number of handled PLCs per program which means that how many PLCs are handled in one program. The fourth feature indicates the scalability whether yes or no to scale the program. Lastly, parallel processing feature shows the possibility if the program can run in parallel (Yes) and by contrast (No).

What is interesting in this figure is that the implementation when applying thread and partition, the result is far better than the execution before applying the thread and partition. All things considered, it seems reasonable to consider applying the thread and partition with regard to the development of Apache Kafka and Apache Spark.

## 9. Conclusions

The following conclusion can be drawn from the present study. Many methods can be used to improve the Kafka streaming data. However, the evidence from this studying suggests that using multiple threads and partitions need to be taken into account because of the reliability and efficiency of the system. Having said that, the algorithm to secure data when transmitting, is also essential to consider in this study. With this in mind, the public/private key encryption and decryption play a vital role in terms of data transmission security. Then again, one of the more significant findings to emerge from this study is that the integration of the Hadoop system especially Apache Kafka and Apache Spark enhances the performance and accuracy of data storing, processing, and securing in the manufacturing environment. Overall, in this paper, we considered secured data with respect to only data transmission, since our system has been used for only a single company. We did not consider the security of data stored in the database in the server. We will deal with the issue in the future work, since our system can be extended to several companies. All things considered, we are aware that our research may have some limitations. These results differ from the general published research, it seems that this paper is not only about the streaming and transmitting of data in general, but also the transitional pattern leveraging the Big Data ecosystem and data transmission security, to achieve our main goal of streaming real-time data effectively and efficiently. Lastly, this research has raised many questions in need of further investigation with respect to the security of data stored in the database. Therefore, further studies, which take this transitional pattern and security of data stored in the database into account, will need to be undertaken. 

## Figures and Tables

**Table 1:** Shows measurement performance of multiple threads and partitions which compare to no thread and partition.

Measurement Feature	Without Thread and Partition	With Thread and Partition
Time Consuming (ms)	34,200	1068.6
Number of Deployed Consumer	6	1
Number of Handled PLCs per Program	1	6
Scalable	No	Yes
Parallel Processing	No	Yes
